# Combined With Mefloquine, Resurrect Colistin Active in Colistin-Resistant *Pseudomonas aeruginosa in vitro* and *in vivo*

**DOI:** 10.3389/fmicb.2021.790220

**Published:** 2021-11-26

**Authors:** Xiaodong Zhang, Yining Zhao, Luozhu Feng, Mengxin Xu, Yiru Ge, Lingbo Wang, Ying Zhang, Jianming Cao, Yao Sun, Qing Wu, Tieli Zhou

**Affiliations:** ^1^Department of Clinical Laboratory, The First Affiliated Hospital of Wenzhou Medical University, Wenzhou, China; ^2^Department of Medical Lab Science, School of Laboratory Medicine and Life Science, Wenzhou Medical University, Wenzhou, China

**Keywords:** colistin resistance, *Pseudomonas aeruginosa*, synergistic effect, mefloquine, anti-biofilm

## Abstract

Colistin is a polymyxin antibiotic that is widely used for the treatment of multidrug resistant (MDR) *Pseudomonas aeruginosa* infections, as the last resort. Over the past few years, unreasonable use of antibiotics has resulted in an increase in MDR strains, including colistin-resistant *P. aeruginosa*. The present study aimed to explore the synergistic effects of mefloquine in combination with colistin for the treatment of colistin-resistant *P. aeruginosa in vivo* and *in vitro*. The synergistic effect of the combination of mefloquine and colistin was investigated *in vitro* using checkerboard method, time-killing assay, biofilm formation inhibition test, and biofilm eradication test. The study also explored the synergistic effects of this combination of drugs *in vivo*, using a *Galleria mellonella* infection model. The results for checkerboard method and time killing curve indicated that mefloquine in combination with colistin showed a good antibacterial activity. Furthermore, the combination of these two drugs inhibited biofilm formation and eradicated pre-formed mature biofilms. This synergistic effect was visualized using scanning electron microscopy (SEM), wherein the results showed that the combination of mefloquine and colistin reduced biofilm formation significantly. Further, the application of this combination of drugs to *in vivo* infection model significantly increased the survival rate of *G. mellonella* larvae. Altogether, the combination of mefloquine and colistin showed a good synergistic effect *in vitro* and *in vivo*, and highlighted its potential to be used as an alternative therapy for the treatment of colistin-resistant *P. aeruginosa* infection.

## Introduction

*Pseudomonas aeruginosa* is an opportunistic pathogen that is responsible for an increase in number of infections, including respiratory infections, urinary tract infections, and bacteremia (Herrera-Espejo et al., 2020). Over the past few years, the widespread use and even abuse/misuse of antibacterial drugs has resulted in the emergence of multidrug resistant (MDR) strains, including *P. aeruginosa* (Hazlett et al., 2019). Such a situation poses a great threat to human health worldwide. The Centers for Disease Control and Prevention has identified MDR *P. aeruginosa*, including colistin-resistant *P. aeruginosa*, as one of the most important pathogens that pose a serious threat to human health worldwide (Mikhail et al., 2019).

Colistin, polymyxin E, is a kind of polypeptide antibiotic, which can be used for the treatment of various infectious diseases caused by gram-negative bacteria (El-Sayed Ahmed et al., 2020). Unfortunately, colistin exerts significant side effects, such as nephrotoxicity and neurotoxicity (Nakwan et al., 2019). Thus, devising strategies to lower colistin concentration to reduce these side effects would be very helpful. However, in the recent years, there has been an increase in MDR gram-negative bacteria, including *P. aeruginosa*, owing to which colistin has been re-used in clinics, particularly due to its low drug resistance rate and good antibacterial activity (Gregoire et al., 2017). However, this widespread use of colistin has resulted in the emergence of colistin resistant strains, including colistin-resistant *P. aeruginosa*, whose numbers are increasing rapidly (Stefaniuk and Tyski, 2019). Therefore, there is an urgent need to find an alternative therapy to deal with colistin-resistant *P. aeruginosa* infections (Mikhail et al., 2019).

It is previously known that biofilm formation ability of *P. aeruginosa* is significantly stronger as compared to other bacteria. In many infectious diseases, caused by *P. aeruginosa*, the formation of biofilm is one of the main reasons that is responsible for poor treatment and recurrent infections (Lee and Yoon, 2017; Thi et al., 2020). Importantly, the formation of biofilm makes the bacteria inside the biofilm resistant to antibiotics, complicating the treatment of *P. aeruginosa* infections (Sharma et al., 2014), especially for colistin-resistant *P. aeruginosa*. Therefore, it is important to devise a therapeutic strategy that could assist in the inhibition of colistin-resistant *P. aeruginosa* biofilm formation and eradication of mature biofilms.

Mefloquine is an anti-malarial drug, which is used to treat malaria by killing red blood cell trophoblasts of the malarial parasite (Podoll et al., 2021). Mefloquine is known to exert a good antibacterial effect on gram-positive bacteria, however, its activity on gram-negative bacteria is poor (Hu and Coates, 2021). In a previous study, Hu and Coates (2021) reported that application of mefloquine in combination with colistin showed a synergistic activity against NDM-1, ESBL, and mcr-1 produced by *Escherichia coli* and *Klebsiella pneumoniae*. However, their effect on biofilm formation was not studied. Additionally, antibacterial and anti-biofilm activities of mefloquine/colistin combination against colistin-resistant *P. aeruginosa* remain unknown. The present study evaluated the synergistic effect of mefloquine and colistin on colistin-resistant *P. aeruginosa* through various *in vitro* and *in vivo* experiments.

## Materials and Methods

### Bacterial Isolates and Growth Conditions

In this study, 8 colistin-resistant *P. aeruginosa* clinical strains were isolated from the First Affiliated Hospital of Wenzhou Medical University during 2015–2017, and the same strain from the same patient was eliminated. All species’ identification was performed by using the matrix-assisted laser desorption/ionization time-of-flight mass spectrometry (MALDI-TOF-MS, BioMerieux, France) according to the manufacturer’s instructions. The isolates were frozen in Luria Bertani (LB) (Thermo Fisher Scientific, America) broth medium supplemented with 30% glycerin at −80°C for further research. As a quality control strain, *P. aeruginosa* ATCC27853 was purchased from the National Center for Clinical Laboratories.

### Antibiotics and Solvents

Mefloquine (MFQ) was purchased from Shanghai Yuanye Biotechnology Co., Ltd. (Shanghai, China). Other clinically commonly used antibiotics used in the present study included aztreonam (ATM), ceftazidime (CAZ), cefepime (FEP), imipenem (IPM), meropenem (MEM), ciprofloxacin (CIP), levofloxacin (LVX), gentamicin (GEN), tobramycin (TOB), amikacin (AMK), piperacillin/tazobactam (TZP), piperacillin (PIP), and colistin (COL) (Wenzhou Kangtai Biotechnology Co., Ltd., Zhejiang China). Mefloquine was dissolved in 2.5% dimethyl sulfoxide (DMSO) (Sigma-Aldrich, Saint Louis, United States). Cation-adjusted Mueller-Hinton broth (CAMHB) (Thermo Fisher Scientific) medium was used for antimicrobial susceptibility testing.

### Antimicrobial Susceptibility Testing

The minimum inhibitory concentrations (MICs) of commonly used antibiotics and mefloquine against 8 colistin-resistant *P. aeruginosa* were determined by the microbroth dilution method, as described elsewhere (Andrews, 2001), with slight modifications. Briefly, the bacteria cultured overnight were adjusted to 0.5 McFarland (0.5 McFarland ≈ 1.5 × 10^8^ CFU/mL) in sterile saline water and diluted at 1:100 in CAMHB. Then, 100 μL of the bacterial suspension was added to 96-well microplates to achieve a final bacterial concentration of 7.5 × 10^5^ CFU/mL. Next, the antibiotics were diluted with twofold serial dilutions in their respective solvents (0.25–256 μg/mL). The antibiotic was added to 96-well plates, and the diluted bacterial suspension was mixed. The results were observed after incubation of the plate at 37°C for 16–18 h. The results were interpreted with reference to the latest CLSI. MIC was defined as the lowest concentration of an antibiotic that could completely inhibit the bacterial growth. Each experiment was repeated independently three times.

### Checkerboard Assays

The checkerboard method was employed to evaluate the synergistic effect of mefloquine and colistin, as described previously (Zharkova et al., 2019), albeit with some minor modifications. This assay was performed in a 96-well microplate. First, colistin was used as the drug A for twofold serial dilutions. Mefloquine in varying concentrations was prepared and used as the drug B. Then, the overnight bacterial culture was adjusted to 0.5 McFarland (0.5 McFarland ≈ 1.5 × 10^8^ CFU/mL) in sterile saline water and diluted at 1:100 in CAMHB. We then added 100 μL of the bacterial suspension to a 96-well microplate, which gave a final bacterial concentration of 7.5 × 10^5^ CFU/mL, followed by mixing with drugs A and B. Finally, the 96-well plates were incubated at 37°C for 16–20 h to observe the results. We then evaluated the synergistic effect of mefloquine combined with colistin by using a fractional inhibitory concentration index (FICI). The FICI was calculated using the formula: FICI = (MIC of drug A in the combination/MIC of drug A alone) + (MIC of drug B in the combination/MIC of drug B alone). Interactions were interpreted as follows: synergy for FICI ≤ 0.5; no interaction for 0.5 < FICI ≤ 4; antagonism for FICI > 4 (She et al., 2021). Each test was performed in triplicate.

### Time-Kill Assay

The time-kill assay was performed to further evaluate the synergistic effect of mefloquine and colistin (Fadwa et al., 2021), with some minor modifications. Four groups were established, namely the control group (without drug), colistin monotherapy group, mefloquine monotherapy group, and combined group. Then, 200 μL of 0.5 McFarland bacterial suspension was added to a 50-mL centrifuge tube containing 20 mL of LB broth. The colistin monotherapy group with a final colistin concentration of 0.25–1 μg/mL, the mefloquine monotherapy group with a final mefloquine concentration of 32–64 μg/mL, and the monotherapy concentration of the corresponding strain was added to the combined group. The 50-mL centrifuge tube was then shaken at 200 rpm at 37°C. The bacterial counts were enumerated on LB agar plates at 0, 2, 4, 6, 12, and 24 h. According to the growth rate of bacteria, appropriate dilution concentration was prepared, and 100 μL of the bacterial suspension was taken at each time point for counting purpose. The bactericidal action was defined as a ≥ 3 log_10_ reduction in CFU/mL at 24 h. The synergistic effect was defined as a ≥ 2 log_10_ reduction in CFU/mL for the combination in comparison to its most active agent after 24 h (Fadwa et al., 2021). All studies were conducted in duplicate.

### Biofilm Formation Inhibition Test

The effect of mefloquine combined with colistin on biofilm formation was studied after crystal violet staining (Djordjevic et al., 2002), albeit with some minor modifications. First, fresh single colonies grown on blood plates were shaken overnight in 3-mL LB broth medium at 37°C. After that, sterile normal saline was used to adjust the turbidity of the strains to 0.5 McFarland, and 1:100 diluted culture in LB broth before being added to the 96-well plate. Mefloquine and colistin were then added to the 96-well plate at a final concentration of 16–64 μg/mL and 0.5–1 μg/mL, respectively, in combination and alone. The 96-well plates were then incubated at 37°C for 24 h. We washed the 96-well plate thrice with sterile phosphate buffered saline (PBS) (Sigma–Aldrich, Milan, Italy) to remove the planktonic bacteria. After the plate dried naturally at room temperature, 1% crystal violet (Beijing Solarbio Biotechnology Co., Ltd., China) was added and the plate was incubated at 37°C for 15 min. After staining, the 96-well plates were cleaned with sterile PBS. After washing, the 96-well plates were naturally dried and 200 μL of 95% ethanol + 5% acetic acid was added to dissolve crystal violet. The dissolved crystal violet was then transferred to a new 96-well plate. Biofilm biomass was determined by measuring the absorbance at 595 nm. All tests were performed in triplicate in at least three independent experiments.

### Preformed Mature Biofilm Eradication Assays

We evaluated the eradication effect of mefloquine combined with colistin on mature biofilm as suggested elsewhere (She et al., 2020), with minor modifications. First, fresh single colonies were picked from a blood plate and shaken overnight in a 3-mL LB broth medium at 37°C. The resultant bacterial suspension was adjusted to 0.5 McFarland with sterile saline water. Then, 0.5 McFarland bacterial suspension was diluted 1:100 in LB broth and incubated in a 96-well plate for 24 h. After incubation, mefloquine and colistin were added to the 96-well plates at a final concentration of 128 and 8 μg/mL alone or in combination. The 96-well plates were then incubated at 37°C for 24 h. The eradication of biofilm by mefloquine in combination with colistin was determined as described previously.

### Scanning Electron Microscope

SEM was used to further study the inhibitory effect of mefloquine/colistin combination on biofilms (Zhang et al., 2020), with minor modifications. In the SEM experiment, TL2314 was selected as the experimental strain. We conducted experiments in sterile cell culture on 6-well plates (NEST Biotechnology Co., Ltd., China). The control group, colistin monotherapy group, mefloquine monotherapy group, and combination group were accordingly established. First, sterile cover slides (lot number: NO.10211818C, CITOGLAS Co., Ltd., China) were placed in a 6-well plate to provide a surface for biofilm formation, to which 100 μL of 0.5 McFarland bacterial suspension was added to the 6-well plate. Then, 1,900 μL of the LB broth was added to the control group, LB broth with a final concentration of 0.5 μg/mL colistin or 64 μg/mL mefloquine was added to the monotherapy group, and LB broth containing a final concentration of 0.5 μg/mL colistin and 64 μg/mL mefloquine was added to the combined group and incubated at 37°C for 24 h. Later, the cover glass was placed in a sterile 6-well cell culture plate containing 2.5% glutaraldehyde, fixed at 4°C for 4 h, and then dehydrated with a series of gradient concentrations (30, 50, 70, 90, and 100%) of ethanol for 10 min. The final samples were dried at room temperature and then observed by SEM (S-3000N, Japan).

### *In vivo* Synergy in the *G. mellonella* Infection Model

We evaluated the synergistic effect of mefloquine and colistin by calculating the survival rate using a *G. mellonella* infection model *in vivo* (Tsai et al., 2016; Grygorcewicz et al., 2020), with minor modifications. Only the evenly uniformly milky white larvae of *G. mellonella* weighing about 250–300 mg were selected for the experiment. TL1671 and TL2314 were used as the experimental strains in this experiment. Briefly, first, a single colony was selected from the blood plate and adjusted to 0.5 McFarland. TL1671 was diluted to 1.5 × 10^2^ CFU/mL and TL2314 to 1.5 × 10^3^ CFU/mL. The following four groups were established: control group, mefloquine monotherapy group, colistin monotherapy group, and combination group. Then, 10 μL of the bacteria suspension was injected into the rear left proleg of *G. mellonella* by using a microinjector. The negative control group was only injected with sterile saline water. The positive control group was injected with a corresponding concentration of bacterial suspension and sterile saline water. The colistin monotherapy group was injected with bacterial suspension and colistin (colistin 0.5, 1, and 2 μg/mL × 7). The mefloquine monotherapy group was injected with the bacterial suspension and mefloquine (64 μg/mL × 7). The combination group was injected with the bacterial suspension. The combined concentrations of mefloquine and colistin were 0.5 + 64 μg/mL × 7, 1 + 64 μg/mL × 7, 2 + 64 μg/mL × 7, respectively. All groups were injected with bacterial suspension 2 h before the drug injection. The larvae were then placed at 37°C to record the 7-day survival rate. The death of *G. mellonella* larvae was determined mainly by recording changes in their skin color and non-response to stimuli. Kaplan-Meier analysis and log-rank test were performed to analyze the survival rate of *G. mellonella* larvae. All experiments were conducted in triplicate.

### Statistical Analysis

Data were expressed as mean ± standard deviation of at least three independent trials. Significance was determined by using two-sample *t*-test, log-rank test and mentioned as *P* < 0.05 (noted with^∗^), *P* < 0.01 (noted with^∗∗^), and *P* < 0.001 (noted with^∗∗∗^). Statistical analyses were performed using the Graph Pad Prism 8.0 statistical software.

## Results

### Antimicrobial Susceptibility Assay

The MICs for common antibiotics used in clinics for the treatment of eight colistin-resistant *P. aeruginosa* strains are listed in Table 1. Most of these strains were characterized by MDR phenotypes. In fact, most of the strains showed different degrees of resistance toward aminoglycosides, fluoroquinolones, β-lactam, and polypeptide antibiotics. Among the tested anti-bacterial agents, the resistance rate of these strains toward TZP, PIP, IPM, and MEM reached more than 75%. The sensitivity toward AMK and FEP was recorded to be the highest, with a resistance rate of 12.5%. MICs of colistin toward all strains were recorded to be in the range of 4–128 μg/mL. MIC for mefloquine was recorded to be > 256 μg/mL for all strains, which indicated that mefloquine exhibited no antibacterial activity against these eight colistin-resistant *P. aeruginosa* strains.

**TABLE 1:** MIC value of colistin-resistant clinical isolates against commonly used clinical antibiotics and mefloquine.

**Strains**	**Antibiotics**	**ATM**	**CAZ**	**FEP**	**IPM**	**MEM**	**CIP**	**LVX**	**GEN**	**TOB**	**AMK**	**TZP**	**PIP**	**COL**	**MFQ**
	**Breakpoint (S-R)**	**8–32**	**8–32**	**8–32**	**2–8**	**2–8**	**0.5–2**	**1–4**	**4–16**	**4–16**	**16–64**	**16/4–128/4**	**16–128**	**2–4**	
ATCC 27853		4	4	2	4	0.5	0.5	4	1	1	2	8/4	4	2	>256
TL-1671		8^S^	4^S^	8^S^	2^S^	0.5^S^	0.25^S^	1^S^	2^S^	1^S^	8^S^	128/4^S^	8^S^	32^R^	>256
TL-1736		8^S^	32^R^	2^S^	16^R^	256^R^	1^I^	1^S^	32^R^	8^I^	32^I^	>256/4^R^	256^R^	16^R^	>256
TL-1744		32^R^	32^R^	16^I^	16^R^	16^R^	32^R^	8^R^	>128^R^	32^R^	≤ 2^S^	256/4^R^	128^R^	64^R^	>256
TL-2314	MIC(μg/mL)	16^I^	32^R^	16^I^	4^I^	4^I^	0.5^S^	2^I^	8^I^	2^S^	32^I^	128/4^R^	256^R^	8^R^	>256
TL-2917		32^R^	16^I^	16^I^	16^R^	256^R^	0.25^S^	2^I^	8^I^	8^I^	4^S^	256/4^R^	256^R^	16^R^	>256
TL-2967		128^R^	16^I^	32^R^	16^R^	128^R^	8^R^	16^R^	8^I^	8^I^	16^S^	256/4^R^	256^R^	4^R^	>256
TL-3008		4^S^	2^S^	4^S^	16^R^	8^R^	0.5^S^	1^S^	16^R^	4^S^	32^I^	16/4^S^	8^S^	64^R^	>256
TL-3086		128^R^	16^I^	16^I^	>128^R^	128^R^	16^R^	8^R^	>128^R^	128^R^	>128^R^	256/4^R^	128^R^	>128^R^	>256

*MIC, Minimum inhibitory concentration; ATM, aztreonam; CAZ, ceftazidime; FEP, cefepime; IPM, imipenem; MEM, meropenem; CIP, ciprofloxacin; LVX, levofloxacin; GEN, gentamicin, TOB, tobramycin; AMK, amikacin; TZP, piperacillin/tazobactam; PIP, piperacillin; MFQ, mefloquine and COL, colistin. S, Susceptible; I, intermediate; R, resistance.*

### Checkerboard Assays

The synergistic effect of application of 0.125–2 μg/mL of colistin in combination with 4–32 μg/mL mefloquine was evaluated against colistin-resistant *P. aeruginosa*. As reported in Table 2, the combination of mefloquine with colistin showed synergistic activity against all strains, with FICI < 0.5. When colistin was combined with mefloquine, MIC values for colistin showed a significant reduction, by 8–64 times. In fact, combination of colistin resulted in a significant change in the sensitivity of all strains toward colistin, wherein drug-resistant phenotype changed to sensitive phenotype.

**TABLE 2:** FICI value for colistin/mefloquine combinations against colistin-resistant *Pseudomonas aeruginosa* strains.

**Strains**	**MIC of Monotherapy (μg/mL)**		**MIC of combination (μg/mL)**		**FICI**	**Interpretation**
	**COL**	**MFQ**	**COL**	**MFQ**		
TL-1671	32	>256	1	4	<0.047	Synergy
TL-1736	16	>256	1	32	<0.188	Synergy
TL-1744	64	> 256	1	8	<0.049	Synergy
TL-2314	8	>256	0.125	16	<0.078	Synergy
TL-2917	16	>256	0.25	16	<0.078	Synergy
TL-2967	4	>256	0.5	16	<0.188	Synergy
TL-3008	64	>256	2	16	<0.094	Synergy
TL-3086	>128	>256	2	16	<0.078	Synergy

*FICI, fractional inhibitory concentration index; MFQ, mefloquine and COL, colistin.*

### Time-Kill Assay

**FIGURE 1:**
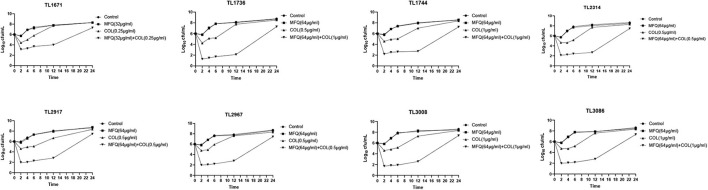
Time kill curve result diagram. Time-killing curves of colistin and mefloquine alone or in combination against Colistin-resistant *P. aeruginosa*. Mefloquine (MFQ) and colistin (COL).

As shown in Figure 1, time killing curve was used to further verify the synergistic effect of mefloquine and colistin on eight colistin-resistant *P. aeruginosa* strains. The drug concentrations used for time-kill curve were derived from the results of checkerboard assay, with FICI < 0.5. The concentrations of mefloquine that were selected included 32 and 64 μg/mL, and the concentrations of colistin were 0.25, 0.5, and 1 μg/mL. When compared with control group, monotherapy group showed little or no time-dependent bactericidal activity, as bacterial load did not decrease. In the first 12 h, mefloquine in combination with colistin significantly reduced the growth of colistin-resistant *P. aeruginosa*, by more than 3 log_10_ (CFU/mL), as compared to control group and monotherapy group. However, the inhibition effect weakened after 12 h, as the bacteria started to regrow at that time.

### Effects of the Combination of Colistin With Mefloquine on Biofilm Formation

**FIGURE 2:**
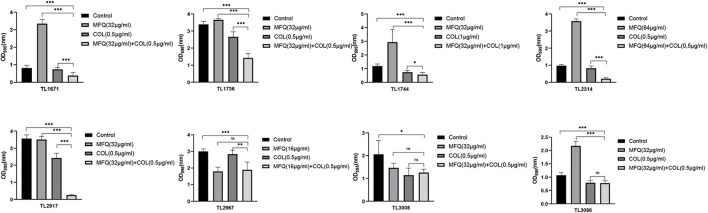
Inhibitory effect of mefloquine combined with colistin on biofilm formation of *Pseudomonas aeruginosa*. The selected drug concentration was derived from the Checkerboard method with FICI < 0.5. *P* < 0.05 (^∗^), *P* < 0.01 (^∗∗^), and *P* < 0.001 (^∗∗∗^) analyzed by Student’s *t*-test. Mefloquine (MFQ) and colistin (COL).

Next, the effects of mefloquine and colistin alone or in combination on the formation of biofilms of *P. aeruginosa* were studied. As shown in Figure 2, mefloquine combined with colistin inhibited biofilm formation in most of the strains (5/8), as compared to monotherapy group and control group (P < 0.05). Additionally, biofilm formation was found to be inhibited in other strains in case of combination group, when compared with the control group, however, there was no difference when compared with monotherapy group. The synergistic concentration for each strain was derived from checkerboard method.

**FIGURE 3:**
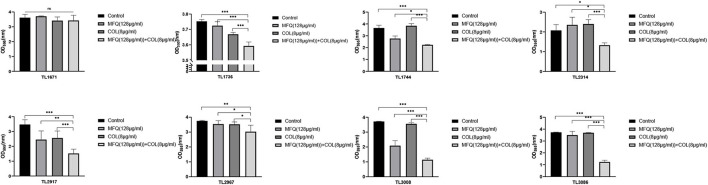
Eradication effect of mefloquine combined with colistin on *Pseudomonas aeruginosa* mature biofilm. The selected drug concentration was derived from the Checkerboard method with FICI < 0.5. *P* < 0.05 (*), *P* < 0.01 (**), and *P* < 0.001 (***) analyzed by Student’s *t*-test. Mefloquine (MFQ) and colistin (COL).

The study also investigated whether mefloquine combined with colistin could eradicate preformed mature biofilms. The results of the same are shown in Figure 3. Mefloquine combined with colistin eradicated biofilms for most of the strains (7/8), when compared with control and monotherapy groups (*P* < 0.05).

Altogether, these results suggested that the combination of mefloquine and colistin could inhibit biofilm formation of *P. aeruginosa*, and also eradicated mature biofilm.

### Scanning Electron Microscopy

**FIGURE 4:**
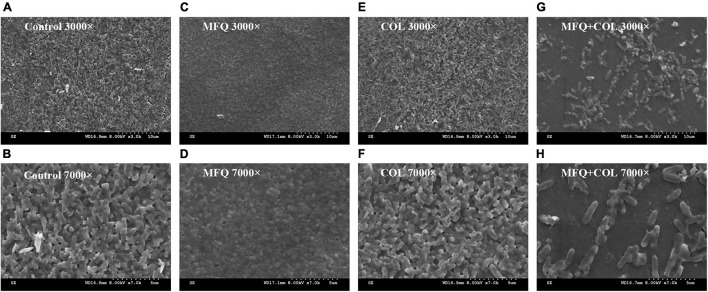
Scanning electron microscopy image. TL2314 was selected as the experimental strain. **(A)**: LB broth control group, 3000×; **(B)**: LB broth control group, 7000×; **(C)**: mefloquine single group, 3000×; **(D)**: mefloquine single group, 7000×; **(E)**: colistin single group, 3000×; **(F)**: colistin single group, 7000×; **(G)**: mefloquine combined with colistin group, 3000×; **(H)**: mefloquine combined with colistin group, 7000×. Mefloquine (MFQ) and colistin (COL).

The inhibitory effect of mefloquine combination with colistin on biofilm formation for colistin-resistant *P. aeruginosa* was visualized using SEM. The results of the same are shown in Figure 4. SEM images showed that biofilms for control group, mefloquine monotherapy group (64 μg/mL), and colistin monotherapy group (0.5 μg/mL) were not damaged, when observed at 3,000 and 7,000 × magnification, wherein the cells were characterized by complete morphology and high density. When compared with control group and monotherapy group, the combined group exhibited more and significant changes, which were mainly manifested at 7,000 × magnifications. In particular, biofilm structure was greatly damaged, and the original, complete, and tightly adhered biofilm was found to be completely destroyed.

### *In vivo* Treatment Verification

**FIGURE 5:**
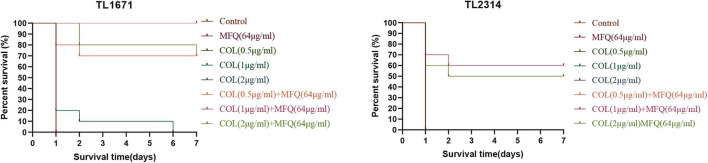
Survival rate of *G. mellonella*. We selected TL1671 and TL2314 as the experimental strains, and recorded the survival rate of *G. mellonella* in 7 days. Mefloquine (MFQ) and colistin (COL).

The efficacy of mefloquine in combination with colistin was further validated *in vivo* by recording survival rates in a model of *G. mellonella* infection. The results of the same are shown in Figure 5. TL1671 and TL2314 were randomly selected as experimental strains. For control group, mefloquine monotherapy group, and colistin monotherapy group, almost no survival was observed in TL1671 within 24 h. However, mefloquine combination with colistin maintained a survival rate of > 70%, after 168 h of the treatment. For the combination of 1 μg/mL of colistin and 64 μg/mL of mefloquine, the survival rate for *G. mellonella* reached 100%, after 168 h (*P* < 0.05). In case of TL2314, control group, monotherapy group, and combination of 0.5 μg/mL of colistin and 64 μg/mL of mefloquine showed no survival after 24 h. The survival rate for *G. mellonella* for two combinations of colistin (1 and 2 μg/mL) and mefloquine (64 μg/mL) reached > 50% (*P* < 0.05).

## Discussion

In the recent years, widespread use and even abuse/misuse of antibacterial drugs has resulted in emergence of various MDR strains. In fact, there has been a rapid increase in the number of such strains. The emergence of MDR bacteria has been shown to be related to higher morbidity and mortality. In fact, it acts as a great economic burden on the healthcare system (Medina and Pieper, 2016), and thus poses a great threat to global public health (Cerceo et al., 2016). *P. aeruginosa* is no exception, and it is known to exhibit MDR phenotypes. Lack of effective antibacterial drugs, slow development of new antibacterial drugs, and high cost of development encourage the rejuvenation of old-antibiotics, such as colistin (Coates et al., 2020), which is often used as the last line of defense antibiotics. The widespread use of colistin has resulted in the appearance of colistin-resistant *P. aeruginosa*. In fact, the number of colistin-resistant strains has increased significantly in the last few years. Importantly, the emergence of colistin-resistant strains would eventually result in failure to control colistin-resistant *P. aeruginosa* infection by clinicians, thereby posing a serious threat to public health. Thus, there is immediate requirement to devise effective treatment strategies to combat *P. aeruginosa* infection. Antibiotic combination therapy might prove to be a good choice in such cases.

For the past few years, several studies reported that the combination of non-antibacterial drugs and colistin exhibited significant synergistic effect on various MDR strains (Vestergaard and Ingmer, 2019; Zhang et al., 2019). The present study evaluated antibacterial and anti-biofilm effects of mefloquine combination with colistin against colistin-resistant *P. aeruginosa*. Mefloquine is a quinoline compound, which is primarily used to treat malaria. A previous study explored and reported antibacterial effects of mefloquine on *Staphylococcus aureus* and *Escherichia coli*. The results showed that mefloquine exerted effect on *Staphylococcus aureus* only at high concentrations, while no antibacterial activity was detected against *Escherichia coli* (Podoll et al., 2021). Another study reported that mefloquine combination with colistin exerted synergistic effects on NDM-1, ESBL, and mcr-1 produced by *Escherichia coli* and *Klebsiella pneumoniae* (Hu and Coates, 2021). However, no previous study explored antibacterial and anti-biofilm effects of mefloquine combination with colistin against colistin-resistant *P. aeruginosa*. The present study evaluated the synergistic effect of mefloquine combination with colistin against colistin-resistant *P. aeruginosa in vitro* and *in vivo*.

First, the synergistic activity of mefloquine and colistin against eight colistin-resistant *P. aeruginosa* strains was verified *in vitro*. Checkerboard method showed that the combination of mefloquine and colistin exhibited a good synergistic effect, wherein FICI values were recorded in the range of 0.047–0.188, which were much lower than 0.5. Since mefloquine alone exhibits little anti-bacterial activity, combination therapy might have delayed the emergence of bacterial resistance. The results for time-killing assay showed that the combination of mefloquine and colistin exerted a significant inhibitory effect on *P. aeruginosa* growth during first 12 h, when compared with most effective single drug group. However, after 12 h, the inhibitory effect weakened, and *P. aeruginosa* growth resumed. This result is inconsistent when compared with the findings of Hu and Coates (2021). It was hypothesized that this might be attributed to either difference in the strains, or there might be persistent bacteria that might have resulted in the regrowth of bacteria after 12 h (Zhang et al., 2021). Therefore, it is necessary to further investigate the causes for this weakened synergistic effect of mefloquine combination with colistin, after 12 h. This result also suggested that if the combinatorial treatment of mefloquine and colistin is used for clinical treatment of *P. aeruginosa* infection, the duration of administration might be shortened.

Quorum sensing (QS) is known to promote biofilm formation (Saipriya et al., 2020). Biofilms are mainly composed of proteins, polysaccharides, nucleic acids, and lipids. The formation of biofilms makes bacteria resistant to antibiotics, rendering most antibiotics powerless to treat bacterial infections (Brackman and Coenye, 2015). At the same time, biofilm is known to be a part of virulence factors (Wu et al., 2020). The formation of biofilms makes many infectious diseases incurable. In fact, it is one of the main causes for recurrent infections (Hoiby et al., 2010). Therefore, the present study also assessed anti-biofilm effects of mefloquine and colistin. The results for biofilm formation inhibition test and eradication test showed that mefloquine combination with colistin could inhibit biofilm formation and also eradicated biofilm for most of *P. aeruginosa* strains. Mefloquine is an anti-malaria drug. Wong et al. (2017) reported that mefloquine is a protein synthesis inhibitor, and its main mechanism of action involves binding to 80S ribosomes of *Plasmodium falciparum* to inhibit protein synthesis, thereby exerting anti-malarial effect. It was hypothesized that the synergy of mefloquine and colistin might be attributed to the fact that colistin changes the permeability of cell membrane, while mefloquine can easily enter bacteria to inhibit protein synthesis, thereby resulting in a good antibacterial effect. The results for biofilm formation inhibition experiment showed that mefloquine monotherapy promoted biofilm formation, while combination of mefloquine with colistin inhibited biofilm formation (Figure 2). It was suspected that under the action of colistin, mefloquine could inhibit the synthesis of biofilm protein, and thus destroyed the structure of biofilm, as observed in SEM micrographs (Figure 4). SEM micrographs showed that biofilm structure was significantly damaged in case of combined treatment group, while it was found to be intact for control group and monotherapy group. It is also possible that the combination of mefloquine and colistin inhibits the expression of QS system, thus inhibiting the formation of biofilm. These results are encouraging and suggested that mefloquine and colistin could be used to inhibit biofilm formation at an early stage and also to eradicate biofilms at a later stage.

The efficacy of mefloquine combination with colistin was further validated *in vivo* using infection model for *G. mellonella*. *In vivo* studies showed that mefloquine combination with colistin increased the survival rate of *G. mellonella*. In the past few decades, colistin use was abandoned due to its serious side effects, such as nephrotoxicity and neurotoxicity (Fiaccadori et al., 2016). The combination of mefloquine and colistin could significantly reduce the amount of colistin, thereby reducing the side effects caused by colistin.

In this study, the combination of mefloquine and colistin exerted a good synergistic effect, as established using various *in vitro* and *in vivo* experiments. However, the only deficiency was that the bacteria showed regrowth after 12 h of combined treatment with mefloquine and colistin, as observed in time-killing curve. It was hypothesized that the bacterial regrowth occurred 12 h after the administration of mefloquine combination with colistin, possibly due to the difference between the strains or the possible generation of persistent bacteria. The novelty of this study lies in the fact that the combination of mefloquine and colistin could not only inhibit the formation of colistin-resistant *P. aeruginosa* biofilms, but it also removed mature biofilms. Subsequently, the reasons for regeneration of *P. aeruginosa* after 12 h of combined action of mefloquine and colistin must be explored in the future. Additionally, future studies must explore the underlying mechanism responsible for anti-biofilm role of the combination of mefloquine and colistin.

## Conclusion

The present study is first to report the synergistic activity of colistin in combination with mefloquine against colistin-resistant *Pseudomonas aeruginosa*. Various *in vitro* and *in vivo* experiments proved that mefloquine and colistin exerted synergistic effects on colistin-resistant *Pseudomonas aeruginosa*. Mefloquine in combination with colistin restored colistin activity, which was encouraging. Altogether, the findings of the present study highlighted the suitability of this combinatorial drug therapy to be used as an alternative therapy for infections caused by colistin-resistant bacteria.

## Data Availability Statement

The raw data supporting the conclusions of this article will be made available by the authors, without undue reservation.

## Author Contributions

XZ conducted the experiments, analyzed the data, and wrote the manuscript. YiZ, LF, and MX participated in the experiments. LW, YG, and YZ participated in the analysis of results. JC and YS participated in the analysis of results. TZ and QW helped design the study. All authors contributed to the article and approved the submitted version.

## Conflict of Interest

The authors declare that the research was conducted in the absence of any commercial or financial relationships that could be construed as a potential conflict of interest.

## Publisher’s Note

All claims expressed in this article are solely those of the authors and do not necessarily represent those of their affiliated organizations, or those of the publisher, the editors and the reviewers. Any product that may be evaluated in this article, or claim that may be made by its manufacturer, is not guaranteed or endorsed by the publisher.
